# Managing Game-Related Conflict With Parents of Young Adults With Internet Gaming Disorder: Development and Feasibility Study of a Virtual Reality App

**DOI:** 10.2196/22494

**Published:** 2021-01-18

**Authors:** Yu-Bin Shin, Jae-Jin Kim, Hyunji Kim, Soo-Jeong Kim, Hyojung Eom, Young Hoon Jung, Eunjoo Kim

**Affiliations:** 1 Institute of Behavioral Science in Medicine Yonsei University College of Medicine Seoul Republic of Korea; 2 Department of Psychiatry Gangnam Severance Hospital Yonsei University College of Medicine Seoul Republic of Korea

**Keywords:** internet gaming disorder, family conflict, coping behavior, virtual reality

## Abstract

**Background:**

Individuals with internet gaming disorder (IGD) report facing family conflicts repeatedly because of their excessive internet gaming. With recent advancements in virtual reality (VR) technology, VR therapy has emerged as a promising method for the management of various psychiatric disorders, including IGD. Given that several risk and protective factors for young people with addiction can be influenced by their interpersonal context, the potential utility of VR-based apps for managing family conflicts needs to be examined with reference to IGD management. However, few studies have evaluated potential treatment modules related to interpersonal conflict management, such as emotion regulation and taking the perspective of others.

**Objective:**

This preliminary study aims to examine the potential use of a VR-based app in the management of game-related conflicts with parents of young adults with IGD and matched controls.

**Methods:**

In total, 50 young male adults (24 with IGD and 26 controls) were recruited to participate in the study. We developed a virtual room where game-related family conflicts arise. Using this room, participants completed 2 VR tasks that required them to express anger and then implement coping skills (ie, risk/benefit assessment of stopping a game and taking parents’ perspective) to deal with negative emotions in interpersonal conflict situations and to decrease one’s gaming behavior.

**Results:**

The results showed that immersion in our VR app tended to provoke negative emotions in individuals with IGD. In addition, after a risk/benefit assessment of stopping a game, the response of stopping a game immediately increased significantly in the IGD group, suggesting that patients’ gaming behavior could be changed using our VR program. Furthermore, in individuals with IGD, longer gaming hours were associated with a lower level of perceived usefulness of the coping skills training.

**Conclusions:**

The findings of this study indicate that our VR app may be useful for implementing more desirable behaviors and managing gaming-related family conflicts in individuals with IGD. Our VR app may offer an alternative for individuals with IGD to learn how a vicious cycle of conflicts is developed and to easily and safely assess their dysfunctional thoughts behind the conflicts (ie, perceived unreasonable risks of stopping a game and thoughts acting as a barrier to taking the perspective of others).

## Introduction

### Background

Internet gaming disorder (IGD), deﬁned as the persistent and recurrent use of the internet to engage in games, can lead to significant psychological distress and impairment in daily social functioning [1]. Given that social, behavioral, and neurobiological development continues until young adulthood, addiction in these periods could have a more striking effect on young populations [2,3]. Therefore, intervention and prevention targeting groups at risk for developing gaming-related problems may reduce the burden of the disease and provide lifelong benefits.

With global efforts to inform effective practice, appropriate therapeutic recommendations for IGD are being made [4]. The most commonly reported approach is a combination of various interventions, including cognitive behavioral therapy (CBT) [5,6], motivational enhancement therapy [7], and pharmacotherapy [4,8]. In addition, with recent advancements in virtual reality (VR) technology, the use of VR coupled with CBT has become a viable treatment option for IGD [9]. However, current VR treatments for patients with IGD have been developed in the context of substance abuse treatment, with a focus on aversive conditioning [9,10] and cue exposure [11]. Thus, few studies have evaluated other potential treatment modules, such as emotion regulation and interpersonal conflict management, which are critical for the management of IGD symptoms. Therefore, it is essential to build an evidence base for other specialized modules for individuals with IGD, which can be implemented using VR technology.

In some East Asian countries, as parental influences may continue even into young adulthood [12,13], young adults as well as adolescents are likely to experience game-related family conflicts. Youth with IGD could experience more difficulties related to handling such conflicts because they often exhibit avoidance coping styles, characterized by the denial of problems or their impact on them [14-16]. Thus, when confronted by their parents who ban gaming, they would deny and refuse to discuss their gaming problems with their parents, which can, in turn, contribute to a harmful cycle of problematic gaming [17]. Therefore, there is a need for specific therapeutic recommendations that not only take into account a repetitive pattern of conflict escalation in the family context but also seek to improve problem-solving skills to resolve these conflicts.

Interpersonal conflict management is one of the clinical domains of psychiatric treatment in which VR-based CBT has been applied increasingly [18,19]. In this context, although some researchers have developed anger-provoking VR [20], an intervention for anger management is still scarce. Studies on VR-based CBT have demonstrated the usefulness of VR in learning social skills via role-playing scenarios [21,22]. Such studies introduced exercises for challenging social contexts (eg, a virtual school) in a stepwise manner, thereby requiring patients to master a series of social skills (eg, starting a conversation and recognizing peers’ emotions) within a VR program [23]. Interactive VR, defined as a dyadic interaction that allows feedback from an avatar, is also known to benefit VR-based CBT [24]. Combining interactive VR with role-playing interpersonal scenarios can help individuals with IGD learn coping skills to handle family conflicts.

The VR scenario we chose for training coping skills in family conflict management is based on the risk/benefit assessment of addictive behavior. From the perspective of risk/benefit analysis, it can be assumed that if the perceived benefits of addictive behavior are weighted more heavily than are perceived risks, then people will continue to engage in such behavior. In accordance with this conjecture, previous studies demonstrated that the tendency to weigh risks more than benefits was predictive of risky and gambling behavior [25]. Moreover, recently, a behavioral intervention for cigarette smokers focusing on a personalized analysis of the risk/benefit of quitting (eg, the risks are the increase in negative affect and reduced ability to concentrate, and the benefits are better health, well-being, and finances) was found to be effective in improving smoking cessation outcomes [26]. Most of the relevant studies seem to focus primarily on perception of the general aspects of risks and benefits of addictive behavior, suggesting that this kind of intervention to treat addictive behavior is feasible in clinical settings [27]. As it is more helpful for adolescents/young adults to set a goal that is shortly and proximally framed, focusing on the risks and benefits of harmful behavior within a short time frame could be more effective. As such, our VR content was implemented to create a perception of stopping gaming immediately, focusing on risk-benefit analysis in a short-term framework.

The second scenario for coping skills training dealt with how individuals cope with negative emotions in interpersonal conflict situations. As disputes on gaming time in the family of individuals with IGD could lead to severe conflict or even violence among family members, emotion regulation in such situations seems to be particularly important for individuals with IGD. Previous research has suggested that one type of emotion regulation involves understanding the current situation from the perspective of others [28]. Thus, in this study, conflict situations escalating into violence were reproduced, and participants were asked to apply taking the perspective of their virtual parents.

### Objectives

Given this background, the objective of this study is to examine the potential of a VR-based app designed to help young adults with IGD to manage game-related conflicts with their parents in a sample of individuals with IGD and matched controls. A virtual room, presumed to be the participants’ own, was developed, in which family conflicts were simulated. To determine whether our VR contents were capable of eliciting negative emotions (ie, anger), the participants engaged in a family conflict situation and were then encouraged to express their anger to their virtual parents who banned gaming. As a part of problem solving–focused training, we implemented the following 2 coping skills: evaluating the perceived benefits and risks (or disadvantages) of stopping the game immediately and taking the perspective of parents on their gaming behavior. As preliminary data on the potential role of VR as a promising approach in the management of IGD, we investigated whether patients with IGD expressed greater anger compared with matched controls and whether their response could be changed after experiencing the coping skill training within our VR program. Finally, we tested whether behaviors in response to our VR program were associated with the severity of IGD and other relevant clinical measures (eg, gaming frequency and motivation for changing gaming behavior).

## Methods

### Sample Size (Power)

Power analysis for an independent *t* test was conducted using the G*Power [29] program to determine the necessary sample size with an α of .05, a power of 0.80, a large effect size (f=0.8), and two tails. The results suggested a desired sample size of 26 participants in each group, totaling 52 subjects, to detect the group difference in our dependent measures.

### Recruitment

Young Asian male adults (24 with IGD and 26 controls) were recruited from the local community using web-based advertisements. As men are known to have a higher prevalence of IGD than women and to avoid gender-specific confounding factors related to emotion expression and coping skills affecting the results, only male participants were recruited [30-32]. All participants were interviewed by a clinical psychologist for IGD diagnosis according to the *Diagnostic and Statistical Manual of Mental Disorders, Fifth Edition* [1]. All enrolled participants with IGD were at the time addicted to at least one of the popular internet games in South Korea (eg, Battle Grounds, League of Legends, and Overwatch). For the controls, reviews of history were performed to confirm whether the participants had ever been diagnosed with IGD. In addition, the controls were screened for a family history of psychiatric disorders; no one had a history of any psychiatric illness in first-degree relatives. The exclusion criteria for all participants were current use of psychotropic medication and any history of substance use disorder, neurological or neurodevelopmental disorder, major depressive disorder, bipolar 1 disorder, and psychotic disorder, as determined using the Mini-International Neuropsychiatric Interview [33]. However, 2 participants with depression were included in the IGD group. Ethical approval was obtained from the institutional review board of Yonsei University Gangnam Severance Hospital. Informed consent was obtained from all participants.

### Measures

Estimated full intellectual functioning (IQ) was obtained using the short form of the Wechsler Adult Intelligence Scale-Revised [34]. Internet gaming behavior was measured in terms of frequency of use (eg, hours of use per week). Participants were asked to rate their current gaming craving on a visual analog scale (VAS), which represents the severity of craving on a 100-mm horizontal line, ranging from no craving at all (left side) to extreme craving (right side). The result is a score on a continuous scale, ranging from 0 to 100.

Readiness to change internet gaming behavior was assessed using a modified version of the readiness to change questionnaire [35]. The modified questionnaire contained 12 items on a 5-point scale, ranging from *strongly disagree* (−2) to *strongly agree* (2), with higher scores representing a higher readiness to change. The total score serves as an index of the motivation to stop playing internet games.

The presence questionnaire (PQ) [36] and the simulator sickness questionnaire (SSQ) [37] were used to measure the users’ experience with our system. PQ is a measure of users’ presence in VR, which is a psychological state of *being there*. Presence can enhance users’ active engagement in content, involving their senses and capturing their attention. SSQ assesses simulator sickness resulting from the discrepancy between simulated visual motion and the sense of movement coming from the vestibular system. Simulator sickness is negatively correlated with users’ enjoyment of VR programs.

### Procedure

After each participant was informed of the purpose of the study and they consented to participate, the IQ assessment was conducted, and data on demographic and clinical characteristics were collected. Subsequently, the participants began the VR session. This study was conducted for approximately 20 to 30 min, with each of the 2 scenarios taking about 10 to 15 min. Their time to completion differed depending on how quickly each participant responded to the tasks in each scenario and which responses were chosen.

**Figure 1:**
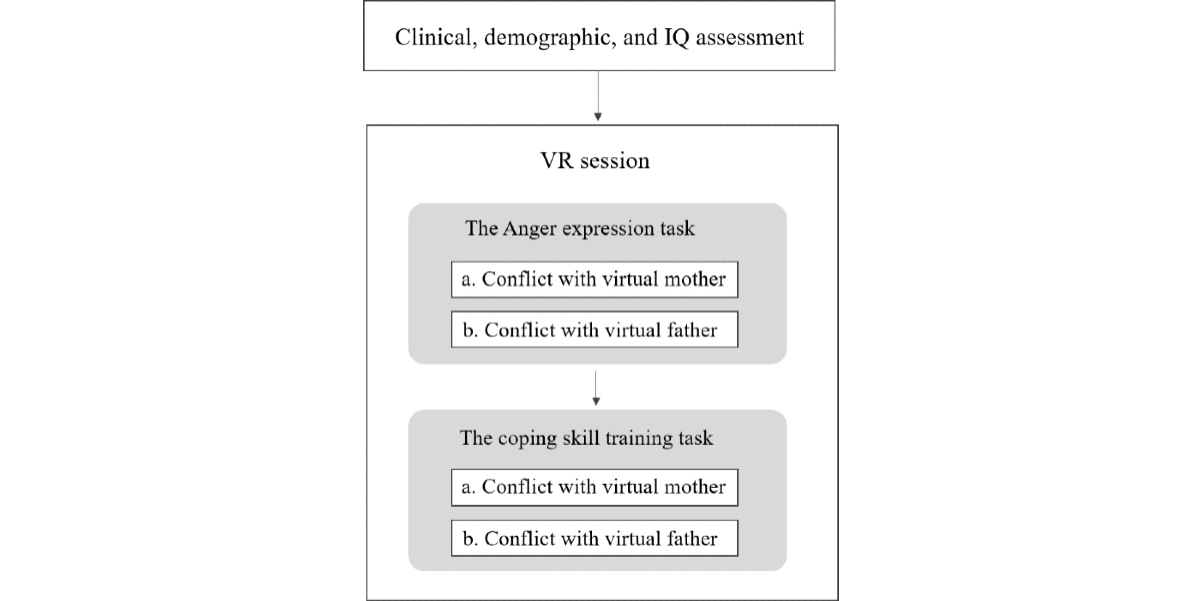
Experimental procedure. The order of tasks on conflicts with the virtual mother and father was counterbalanced. VR: virtual reality.

As shown in Figure 1, all participants began with the anger expression task, but tasks with the virtual mother or father were randomized and counterbalanced among participants to control for order effects.

### Virtual Reality

We developed our own VR scenarios. Our VR scenarios emphasized the development of motivation for change based on the implementation of CBT principles by enabling the participants to access dysfunctional thoughts behind the conflicts (ie, perceived unreasonable risks of stopping a game and thoughts acting as a barrier to perspective-taking) easily.

**Figure 2:**
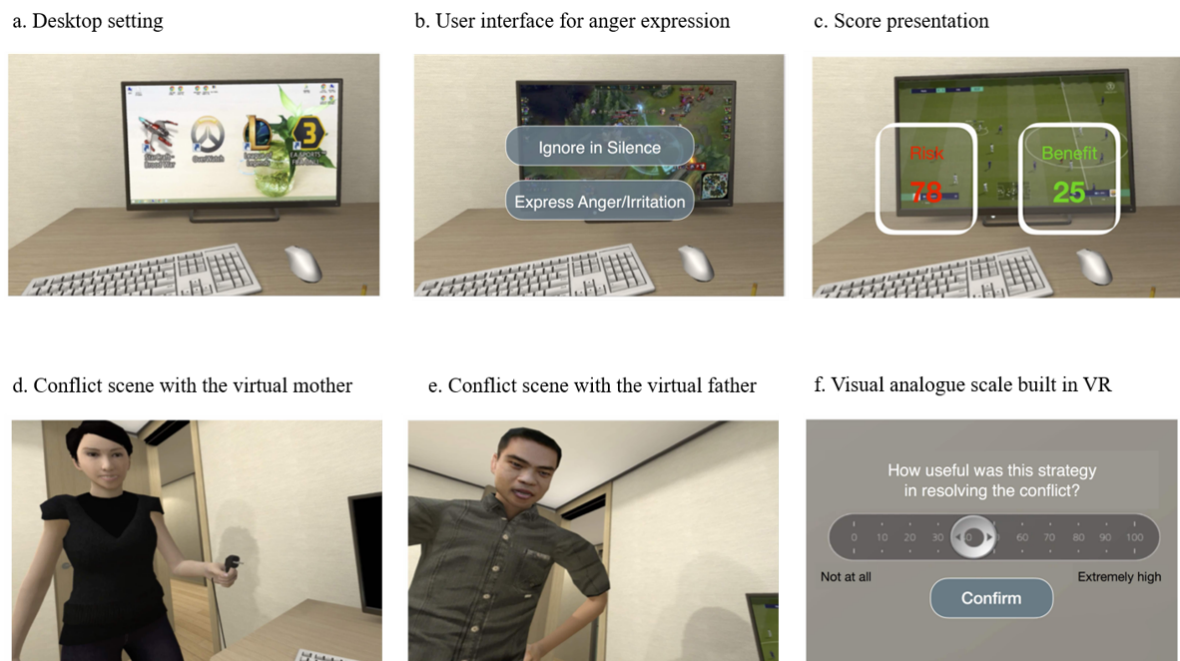
Screenshots of the virtual environment. VR: virtual reality.

**Figure 3:**
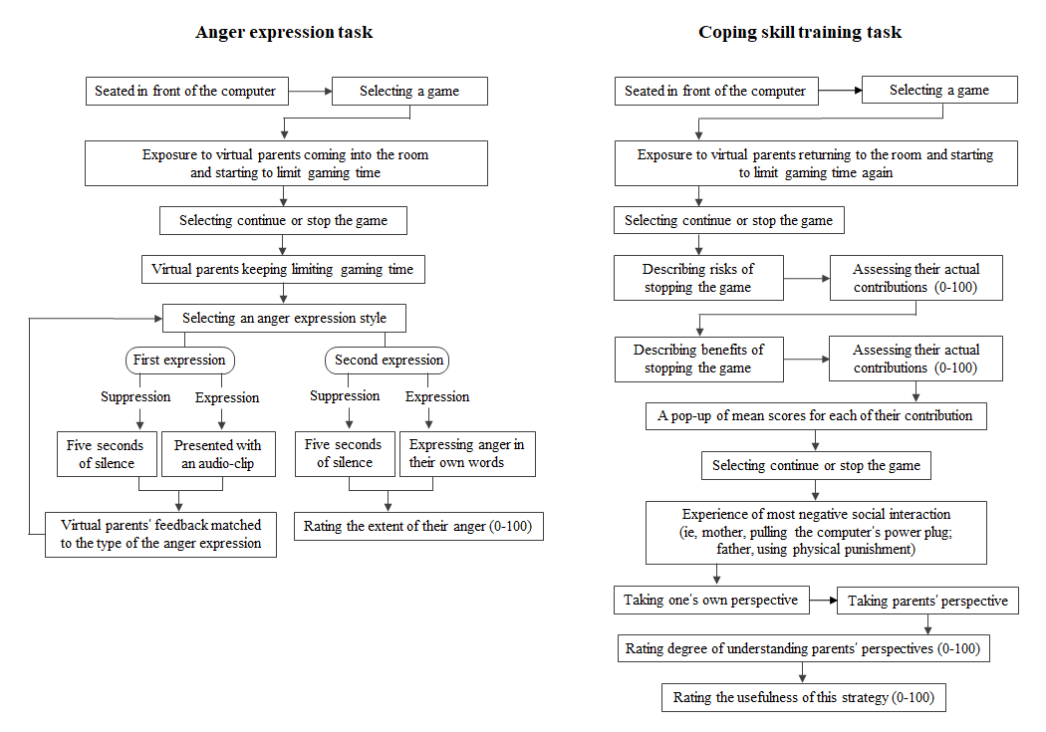
Flowchart of the virtual reality scenario.

Participants were guided through the following 2 sets of VR tasks in a virtual room, presumed to be the participants’ own room: (1) an anger expression task and (2) a coping skill training task (see Figure 2 for screenshots of the virtual environment). Figure 3 presents a flowchart of the scenario. At the beginning of all tasks, participants found themselves seated in front of a monitor displaying internet game icons (eg, StarCraft, Overwatch, League of Legends, and FIFA Online; Figure 2). The participant watched a video clip of their own choice played on the monitor in this VR program. The scenario continued depending on the task content. Every task ended by rating the usefulness of the strategy in resolving the conflict on a VAS built within the VR program (Figure 2).

### Anger Expression Task

In the anger expression task, virtual parents would enter the room and begin compelling the participants to stop the game immediately. Next, the participants were offered a choice of whether to *continue* or *stop* the game. Regardless of which behavior they selected, the scenario would continue with another round of nagging from the parents to stop the game. Participants were then instructed to express their perceived anger about the 2 instances in which they were being forced to end the game. They were offered only a choice-based response format to express their initial anger (ie, suppression: *ignore in silence* or expression: *express anger/irritation*; Figure 2). If they selected *suppression*, the scenario continued to the next scene after 5 seconds of silence. If they selected *expression*, corresponding audio clips of a male actor’s voice were played. Following the completion of the initial expression, parents’ feedback was delivered, the content of which matched the participants’ anger expression response (see Multimedia Appendix 1 for the scripts of the clips). When expressing anger for the second time, participants were allowed to choose the anger expression style and were allowed to express their anger verbally to the parents’ avatars. At the end of each expression, the participants rated the extent of their anger experience on a VAS built within the VR program (0=not at all, 100=extremely high; Figure 2).

### Coping Skill Training Task: Risk/Benefit Assessment and Perspective-Taking Task

In the coping skill training task, the virtual parents returned to the room, asking the participants why they had not stopped the game yet and attempting to limit their gaming time again. Participants were again offered a choice to either *continue* or *stop* the game immediately. Subsequently, they were instructed to employ the 2 coping skills of risk/benefit assessment and perspective-taking. Specifically, they were asked to provide an example of benefits and disadvantages of stopping the game immediately (ie, “What are the benefits/disadvantages of stopping the game immediately? Please describe an example”) and to assess the extent of the effect of each example on them (ie, “How much effect will the example you described have on you?”) using a VAS built within the VR program. They were asked to report as many examples as possible. These items were listed one at a time until they wished to move to the next question. After viewing the mean scores of their effect assessment (Figure 2), they were finally offered the choice of whether to *continue* or *stop* the game immediately. As before, regardless of their choice, the scenario continued with the parent’s pushing the participant to stop the game again. However, this time, the participants experienced the most negative social interaction in the VR situation. For example, the virtual mother turned off the game by pulling the computer’s power plug (Figure 2) or the virtual father used physical punishment by spanking the participant (Figure 2).

For the second coping skill, the participants were asked to talk about the parents’ behavior from their own perspective, followed by talking about it from the parents’ perspective. Subsequently, they rated the extent to which they were able to understand their parents’ perspective using a VAS built within the VR program.

The VR system consisted of a desktop computer (with the Microsoft Windows 10 operating system, an NVIDIA GeForce GTX 970 graphics card, and a 16 GB RAM graphics memory) and an Oculus Rift head-mounted display (Oculus VR; HD resolution of 1080×1200 per eye with a 51.6 diagonal field of view, a 3 degrees of freedom tracker for head rotation, and built-in headphones). The Oculus Touch controller was used for interactions with executable objects and avatars during the VR experience. The microphone built into the VR headset gathered verbal data of users’ self-speech in real time. Three-dimensional virtual environments included a virtual house and appliances using Autodesk 3D max and were integrated with Unity software. User data such as selected responses and selection time, head movement, hand gestures, speech contents, and speaking time were recorded and stored in the main server computer. This system allowed the therapist to track patients’ performance and analyze behavioral information.

### Statistical Analysis

We tested differences between groups on demographic and clinical variables using independent *t* tests. In addition, we conducted repeated measures analysis of variance (ANOVA) to test whether the 2 groups differed in the variables of interest in each VR scenario. To further examine the significant main effects and interactions, we conducted *post hoc* analyses, with Bonferroni correction at *P*<.05. Finally, Pearson correlation coefficients were used to investigate the relationships between the behavioral responses of the virtual environment and clinical variables. Statistical differences were considered significant at *P*<.05. All analyses were conducted using SPSS 23.0 (IBM Corporation).

## Results

### Comparison of Clinical Symptom Measures Between IGD and Control Groups

Table 1 shows the results of a two-tailed independent *t* test between the IGD and control groups in demographic and clinical variables. There were no differences in age, education, and IQ between groups. As expected, the IGD group had higher scores on gaming craving, IGD symptoms, and internet gaming hours per week.

**Table 1:** Demographic and clinical characteristics of participants with internet gaming disorder versus healthy controls.

Characteristics	IGD^a^ (n=24), mean (SD)	Healthy controls (n=26), mean (SD)	*t* test (*df*)	*P* value
Age (years)	21.78 (2.33)	21.65 (1.83)	−0.22 (48)	.83
Education (years)	14.12 (1.19)	14.61 (0.94)	1.62 (48)	.11
IQ	119.54 (10.11)	122.04 (9.72)	0.92 (48)	.36
Gaming craving (visual analog scale)	6.87 (1.46)	0.85 (1.05)	0.41 (48)	<.001
Severity of IGD^b^	7.29 (1.08)	N/A^c^	N/A	N/A
Internet gaming hours per week	50.12 (13.27)	1.99 (1.30)	−18.41 (48)	<.001
Presence Questionnaire	102.29 (16.83)	114.92 (12.93)	2.99 (48)	<.001
Simulator sickness questionnaire	7.45 (7.68)	4.53 (5.73)	−1.53 (48)	.13

^a^IGD: internet gaming disorder.

^b^Severity of IGD was assessed using the Diagnostic and Statistical Manual of Mental Disorders, Fifth Edition, criteria for IGD, providing a total score between 0 and 9.

^c^N/A: not applicable.

For scores on the PQ, there was a significant difference between the IGD and control groups (t_48_=2.99; *P*<.001). As internet games played among participants with IGD have high-quality graphics and animations, the VR environment and interactions might be perceived as less realistic and natural in the IGD group. Regardless of the group difference, the overall mean score was above the midpoint of the scale, indicating that participants experienced a high level of presence in the respective virtual environment.

The average score on the SSQ corresponded to lower levels of simulator sickness than the established norms for the SSQ [37]. An independent *t* test reported no significant difference between the 2 groups in their SSQ scores (t_48_=−1.530; *P*=.13). This shows that both the IGD and control groups had similar VR experiences.

### Anger Expression Style and Intensity of Anger Experience

**Figure 4:**
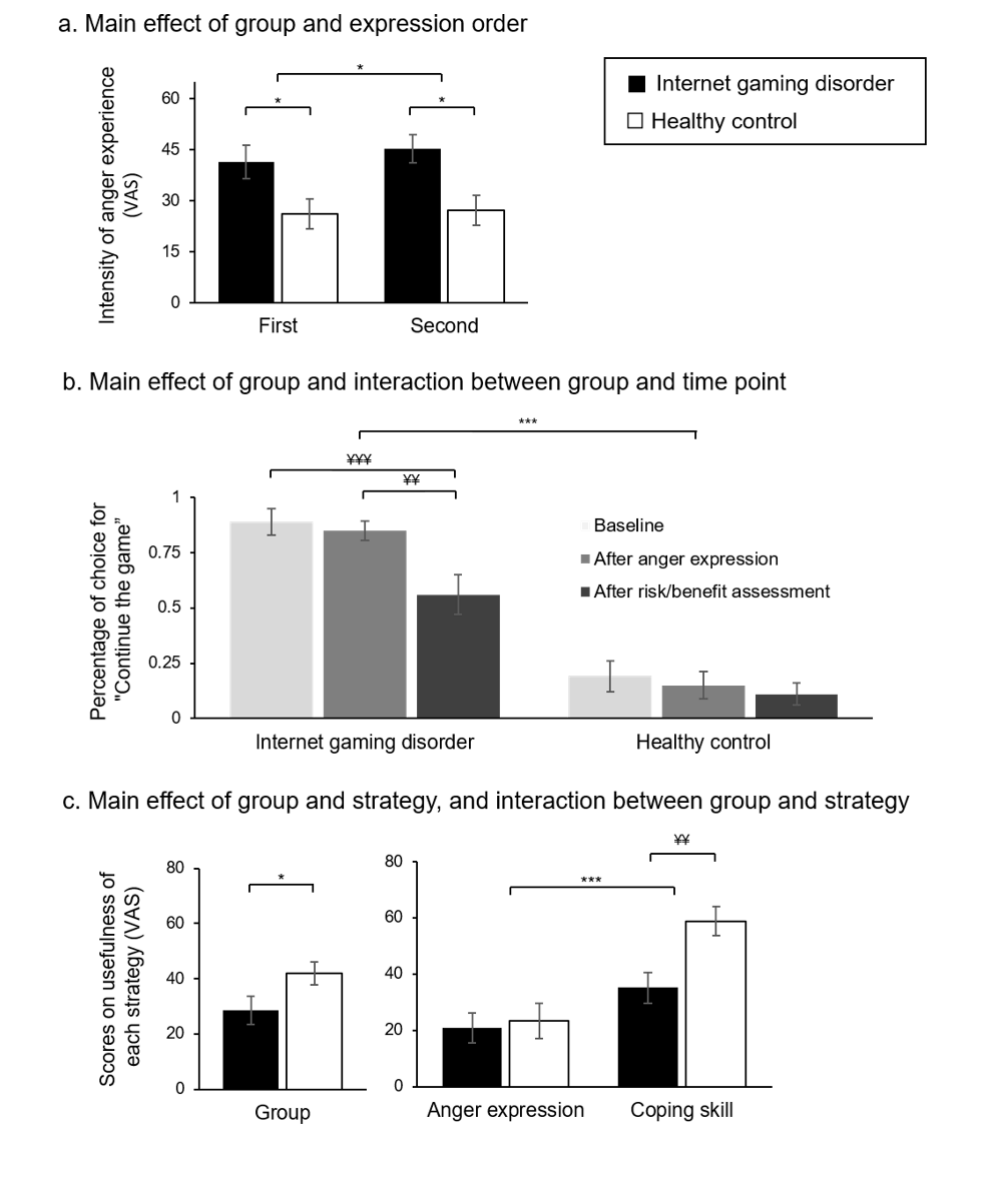
Results of analyses of 3 dependent variables. (a) Mean ratings for the intensity of the anger experience; (b) mean percentage of response choice for “continue the game”; (c) mean ratings for usefulness of each strategy. Error bars represent SE of the mean. *: group or condition effect, *P<.05, **P<.01, ***P<.001; ¥: significant two-way interaction, ¥¥: *P*<.01; ¥¥¥: *P*<.001.

Figure 4 shows the results of a three-way ANOVA of the 2 dependent variables in the anger expression task. The intensity of anger experience revealed a significant main effect of group (*F*_1,48_=8.154; *P*=.006), target (*F*_1,48_=4.278; *P*=.04), and expression order (*F*_1,48_=5.221; *P*=.03; Figure 4). However, no main or interaction effects were observed for the anger expression style. These results indicate that the IGD group experienced higher levels of anger than the control group, the virtual father produced significantly higher anger than the virtual mother, and the second anger expression produced significantly higher anger than the first expression.

### Risk/Benefit Assessment of Stopping the Game Immediately

A 3-way ANOVA on the number of perceived risks (disadvantages) and benefits of stopping the game immediately revealed a significant interaction between group and evaluation type (*F*_1,48_=10.157; *P*=.003), indicating the tendency of the control participants to report significantly more benefits than risks (t_25_=−3.277; *P*=.003). Difference scores (the number of benefit items minus the number of risk items) were used in the correlation analyses. Among individuals with IGD, higher scores, indicating more benefits than risks, were significantly associated with enhanced motivation to change their gaming behavior (*r*=0.488; *P*=.01), as measured by the scores on the readiness to change the questionnaire.

A 3-way ANOVA of the quantified scores on the extent of the effect of the risk-benefit assessment revealed a significant main effect of evaluation type (*F*_1,48_=47.048; *P*<.001) and interaction between group and evaluation type (*F*_1,48_= 23.445; *P*<.001), indicating that benefits had a stronger effect on participants than did risks, and the control group reported a stronger effect of benefits than risks (t_25_=−7.245; *P*<.001).

### Response of Stopping the Game After Each Time Point

A 3-way ANOVA on deciding to stop or continue the game at different time points revealed a significant main effect for group (*F*_1,48_=73.646; *P*<.001), with the IGD group exhibiting a higher tendency to continue the game than the control group, and the main effect for time point (*F*_1,48_=8.490; *P*=.001; Figure 4), indicating a higher tendency to stop the game after the risk/benefit assessment, than that at baseline (mean difference 0.205, SE 0.058; *P*=.003) and after anger expression (mean difference 0.165, SE 0.057; *P*=.02). A significant group and time point interaction was also noted (*F*_1,48_=3.886; *P*=.03), which was caused by the higher tendency of the IGD group to stop the game after the risk/benefit assessment than at baseline (t_23_=3.761; *P*=.001) and after anger expression (t_23_=2.933; *P*=.007).

### Degree of Understanding the Virtual Parents’ Perspective

A 2-way ANOVA revealed a significant main effect for group (*F*_1,48_=17.656; *P*<.001) and target (*F*_1,48_=4.337; *P*=.04). In other words, the control participants exhibited a better understanding of their parents’ perspective than those with IGD, and the participants had a better understanding of the virtual mother’s perspective than that of the virtual father.

### Usefulness of the Conflict-Resolution Strategy

A 3-way ANOVA on ratings for the usefulness of each strategy in resolving conflicts showed a significant main effect for group (*F*_1,48_=5.328; *P*=.03) and strategy (*F*_1,48_=52.667; *P*<.001); the control group evaluated the VR contents as more useful in resolving conflicts (Figure 4), and participants rated the coping skill as more useful in resolving conflicts than anger expression. There was a significant interaction between group and strategy (*F*_1,48_=9.201; *P*=.004), indicating that the control group rated the coping skill as more useful than the IGD group (t_48_=3.697; *P*=.001), but there was no significant difference in anger expression (t_48_=0.372; *P*=.71).

**Figure 5:**
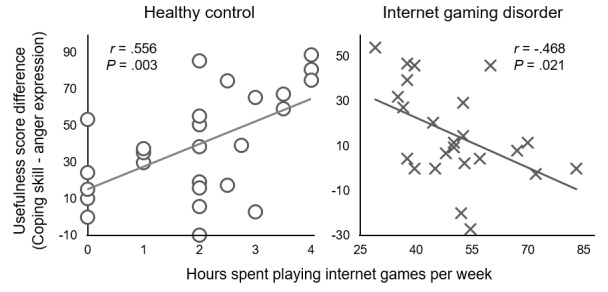
Correlations of mean gaming hours per week with a difference in the usefulness score of strategies.

As presented in Figure 5, in the control group, a higher number of gaming hours was associated with a higher tendency to perceive coping skills as more useful than anger expression (*r*=0.556; *P*=.003). In contrast, in the IGD group, fewer gaming hours were associated with a higher tendency to perceive coping skills as more useful than anger expression (*r*=−0.468; *P*=.02).

## Discussion

### Principal Findings

In this study, we investigated the feasibility of a VR app for managing game-related conflicts with parents in young adults with IGD. The results showed that immersion in our VR program could provoke negative emotions (ie, anger) in individuals with IGD by placing them in interpersonal situations that would require them to manage these conflicts. Moreover, the clinical potential of our VR program was evidenced by the results of the (1) change in participants’ gaming behavior within the program and (2) relationship between patients’ usefulness rating for the implemented coping skills in resolving conflicts and the number of hours spent on gaming.

According to parents of adolescents with IGD, when they attempt to enforce time limits on the game, their children become angry, irrational, and even violent [12]. Thus, anger and violence seem to be common ways to manage such conflicts in families of individuals with IGD. In this context, our VR contents were developed to reproduce such anger-provoking situations in a hierarchical and interactive manner (eg, implementing staged manipulation to increase anger induction, matching the content of virtual parents’ feedback to participants’ anger expression type to simulate dyadic interactions, and allowing participants to express anger via a choice-based response format and verbal response) to increase the resemblance of our VR scenarios to reality. Data from the anger expression task showed that the intensity of the anger experience was higher in individuals with IGD than in control participants, suggesting that family conflict and its emotional response (ie, anger) can be simulated in VR scenarios. In line with this result, the perceived anger after the second expression was stronger than that after the first expression, indicating that the severity of the conflict increased. This experience may closely reflect the nature of conflict regarding time limits on games in families of youth with IGD. Therefore, the use of hierarchical and interactive scenarios for anger induction in VR apps could aid the closer-to-reality simulation of the progression of game-related arguments between family members.

In this study, the VR scenarios allowed participants to consider the benefits and risks of stopping the game immediately as a coping skill for managing family conflicts. The results showed that control participants reported more benefit items than risks. However, no difference was seen in the number of examples of risks and benefits expressed by participants in the IGD group. In line with this result, control participants reported that perceived benefits had a greater influence on them than perceived risks, whereas individuals with IGD did not assign more weight to the effect of benefits than risks. This perception could contribute to their continuous engagement in addictive behavior, as observed in other addictive disorders [25,27]. Hence, this study demonstrates the potential applicability of VR in treating IGD by helping participants to learn the use of risk/benefit assessment of stopping a game.

Our most important finding is that after engaging in assessing the risk/benefit of stopping gaming immediately during repeated exposure to parents’ attempts to limit game time, the IGD group exhibited a higher tendency to choose to stop gaming. As one of the putative mechanisms of CBT to elicit adaptive behaviors is the acquisition of coping and problem-solving skills [38], the results of this study suggest that VR-CBT using risk/benefit assessment to stop gaming could potentially help individuals with IGD to exhibit more desirable behaviors and manage gaming-related family conflicts more effectively.

Taking another person’s perspective is one of the important processes involved in social cognition [39]. Deficits in social cognition may increase social withdrawal and aggression, which might lead to a vicious cycle of substance use [40]. In addition, consistent with the role of empathy in addiction, a previous study reported an association between empathy and IGD symptoms [41]. Similarly, in this study, although participants had the opportunity to talk about the virtual parents’ feelings or thoughts from their perspective, individuals with IGD exhibited a lower understanding of the perspective of the virtual parents compared with control participants. Thus, it can be inferred that impairment of the perspective-taking ability in individuals with IGD may hinder the resolution of game-related conflicts with significant others in real life, which, in turn, may lead them to continue gaming for excessive periods. Together, the findings of this study suggest that our VR content has the potential to be used as a medium for anger management by offering individuals with IGD opportunities to practice taking the perspective of significant others in real-life scenarios and to discuss their thoughts and feelings about difficulties in perspective-taking with clinicians.

After implementing each strategy to manage conflicts with the virtual parents, the participants were instructed to evaluate the overall usefulness of the conflict-resolution strategy employed. Overall, compared with control participants, individuals with IGD evaluated the 2 coping skills as less useful. Moreover, patients with longer gaming hours perceived coping skills as less useful than anger expression in resolving conflicts. This result suggests that IGD individuals with longer gaming hours may not feel the need to engage in more adaptive coping strategies to decrease their gaming hours. Moreover, the association between the usefulness rating of coping skill training in our VR and actual gaming hours indicates that VR parameters may reflect real-life gaming behavior of IGD youths or having a greater tendency to use adaptive coping skills are correlated with less severe symptoms of IGD measured by the gaming hours. Thus, the VR program may benefit young adults with IGD by aiding the assessment of IGD symptomatology. Moreover, given that adaptive coping acts as a buffer against psychological problems, a prior study suggested that a greater tendency to use coping skills such as risk/benefit assessment and perspective-taking of others could mitigate the development of more severe symptoms of IGD [42].

### Limitations

This study has some limitations that warrant future research. The small sample size, sample composition of young Asian male adults, and relatively mild level of IGD severity among our participants limit the generalizability of our findings to the general population. In addition, 2 patients with mild depression were included; the presence of a comorbid condition may be a confounding factor for them. However, the main findings of our study generally remained the same as when excluding participants with comorbidities. Our VR contents still have to be tested in a larger, more heterogeneous sample before its potential role in IGD management can be discussed. Moreover, the present brief and time-limited application of our VR does not suggest that the behavior change achieved could also be applied to real-life situations. Furthermore, behavioral or questionnaire measures were not used to assess the empathic or perspective-taking ability of the participants. In addition, many feedback options were predesigned by the authors, such as the expressions of anger being read out by a male voice in our VR scenario. If participants had been able to verbalize their emotions in their own words, the results of our experiment might have differed. Prior studies have reported that *self-speech* is helpful for self-regulating cognition and behavior [43]. As computer technologies related to voice recognition and acoustic meaning-based speech analysis have developed, speech has also become a viable interaction modality in VR environments. Therefore, a scenario with a self-speech component should be designed to explore this issue in future studies.

In addition, the levels of symptom severity or other behavioral measures in the VR program were based only on subjective reports. Objective physiological markers, such as measurement of psychophysiological reactivity or reports from caregivers regarding participants’ behavioral/emotional problems, would provide additional information. Finally, our preliminary study design did not allow for the validation of the ability of our program to reduce IGD symptoms or increase one’s perspective-taking ability. To further validate the effects of this program, a follow-up study should include a control group comprising individuals with IGD who are not exposed to this VR app.

### Conclusions

We developed a VR program that simulated gaming-related conflicts between young adults and parents and tested its effectiveness in managing problems between family members in an adaptive manner. Considering that the traditional therapeutic approach is dependent on in vivo (real-life) exposure or imagination capabilities of individuals, our VR program may offer an alternative for individuals with IGD to learn how a chronic cycle of conflicts is developed. In addition, it enabled them to access dysfunctional thoughts behind the conflicts (ie, perceived unreasonable risks of stopping a game and thoughts acting as a barrier to perspective-taking) easily and safely. Given that a number of risk and protective factors for young individuals with addiction could be influenced by the family context, our findings suggest the potential use of VR-based apps in the management of family conflicts experienced by individuals with IGD. A clinical trial using VR combined with CBT may shed more light on the effects of learning in VR over time.

## Appendix

Multimedia Appendix 1 Content of participants’ anger expression and virtual parents’ feedback.

## Abbreviations

ANOVA: analysis of variance
CBT: cognitive behavioral therapy
IGD: internet gaming disorder
MET: motivational enhancement therapy
PQ: presence questionnaire
SSQ: simulator sickness questionnaire
VR: virtual reality

## References

[1] American Psychiatric Association (2013). Diagnostic and Statistical Manual of Mental Disorders (DSM-5).

[2] Diamond A (2002). Normal Development of Prefrontal Cortex From Birth to Young Adulthood: Cognitive Functions, Anatomy, and Biochemistry.

[3] Schulenberg JE, Maggs JL (2002). A developmental perspective on alcohol use and heavy drinking during adolescence and the transition to young adulthood. J Stud Alcohol Suppl.

[4] King DL, Delfabbro PH, Wu AM, Doh YY, Kuss DJ, Pallesen S, Mentzoni R, Carragher N, Sakuma H (2017). Treatment of Internet gaming disorder: an international systematic review and CONSORT evaluation. Clin Psychol Rev.

[5] Torres-Rodríguez A, Griffiths MD, Carbonell X (2017). The treatment of internet gaming disorder: a brief overview of the pipatic program. Int J Ment Health Addict.

[6] Young KS (2013). Treatment outcomes using CBT-IA with internet-addicted patients. J Behav Addict.

[7] Shek DT, Tang VM, Lo CY (2009). Evaluation of an Internet addiction treatment program for Chinese adolescents in Hong Kong. Adolescence.

[8] Sancho M, de Gracia M, Rodríguez RC, Mallorquí-Bagué N, Sánchez-González J, Trujols J, Sánchez I, Jiménez-Murcia S, Menchón JM (2018). Mindfulness-based interventions for the treatment of substance and behavioral addictions: a systematic review. Front Psychiatry.

[9] Park SY, Kim SM, Roh S, Soh M, Lee SH, Kim H, Lee YS, Han DH (2016). The effects of a virtual reality treatment program for online gaming addiction. Comput Methods Programs Biomed.

[10] Zhang MW, Ho RC (2017). Smartphone applications for immersive virtual reality therapy for internet addiction and internet gaming disorder. Technol Health Care.

[11] Shin YB, Kim JJ, Kim MK, Kyeong S, Jung YH, Eom H, Kim E (2018). Development of an effective virtual environment in eliciting craving in adolescents and young adults with internet gaming disorder. PLoS One.

[12] Young K (2009). Understanding online gaming addiction and treatment issues for adolescents. Am J Family Therapy.

[13] Fingerman KL, Cheng Y, Kim K, Fung HH, Han G, Lang FR, Lee W, Wagner J (2016). Parental Involvement with College Students in Germany, Hong Kong, Korea, and the United States. J Fam Issues.

[14] Phelps SB, Jarvis PA (1994). Coping in adolescence: empirical evidence for a theoretically based approach to assessing coping. J Youth Adolescence.

[15] Schneider LA, King DL, Delfabbro PH (2017). Maladaptive coping styles in adolescents with internet gaming disorder symptoms. Int J Ment Health Addiction.

[16] Thompson SC (1981). Will it hurt less if I can control it? A complex answer to a simple question. Psychol Bull.

[17] Estevez A, Jauregui P, Lopez-Gonzalez H (2019). Attachment and behavioral addictions in adolescents: the mediating and moderating role of coping strategies. Scand J Psychol.

[18] Parsons S, Mitchell P (2002). The potential of virtual reality in social skills training for people with autistic spectrum disorders. J Intellect Disabil Res.

[19] Tan BL, Lee SA, Lee J (2018). Social cognitive interventions for people with schizophrenia: a systematic review. Asian J Psychiatr.

[20] Brinkman W, Hattangadi N, Meziane Z, Pulditors (2011). Design and Evaluation of a Virtual Environment for the Treatment of Anger. Proceedings of Virtual Reality International Conference.

[21] Kandalaft MR, Didehbani N, Krawczyk DC, Allen TT, Chapman SB (2013). Virtual reality social cognition training for young adults with high-functioning autism. J Autism Dev Disord.

[22] Park KM, Ku J, Choi SH, Jang HJ, Park JY, Kim SI, Kim JJ (2011). A virtual reality application in role-plays of social skills training for schizophrenia: a randomized, controlled trial. Psychiatry Res.

[23] Didehbani N, Allen T, Kandalaft M, Krawczyk D, Chapman S (2016). Virtual Reality Social Cognition Training for children with high functioning autism. Comput Hum Behav.

[24] Rus-Calafell M, Garety P, Sason E, Craig TJK, Valmaggia LR (2018). Virtual reality in the assessment and treatment of psychosis: a systematic review of its utility, acceptability and effectiveness. Psychol Med.

[25] Wickwire EM, Whelan JP, West R, Meyers A, McCausland C, Luellen J (2007). Perceived availability, risks, and benefits of gambling among college students. J Gambl Stud.

[26] McKee SA, O'Malley SS, Salovey P, Krishnan-Sarin S, Mazure CM (2005). Perceived risks and benefits of smoking cessation: gender-specific predictors of motivation and treatment outcome. Addict Behav.

[27] Weinberger AH, Pittman B, Mazure CM, McKee SA (2015). A behavioral smoking treatment based on perceived risks of quitting: a preliminary feasibility and acceptability study with female smokers. Addict Res Theory.

[28] Seehausen M, Kazzer P, Bajbouj M, Prehn K (2012). Effects of empathic paraphrasing - extrinsic emotion regulation in social conflict. Front Psychol.

[29] Faul F, Erdfelder E, Buchner A, Lang AG (2009). Statistical power analyses using G*Power 3.1: tests for correlation and regression analyses. Behav Res Methods.

[30] Chaplin TM (2015). Gender and emotion expression: a developmental contextual perspective. Emot Rev.

[31] Nolen-Hoeksema S, Hilt L (2006). Possible contributors to the gender differences in alcohol use and problems. J General Psychol.

[32] Ko CH, Yen JY, Chen CC, Chen SH, Yen CF (2005). Gender differences and related factors affecting online gaming addiction among Taiwanese adolescents. J Nerv Ment Dis.

[33] Sheehan DV, Sheehan KH, Shytle RD, Janavs J, Bannon Y, Rogers JE, Milo KM, Stock SL, Wilkinson B (2010). Reliability and validity of the mini international neuropsychiatric interview for children and adolescents (mini-kid). J Clin Psychiatry.

[34] Donders J, Warschausky S (2007). Validity of a short form of the WISC-III in children with traumatic head injury. Child Neuropsychol.

[35] Rollnick S, Heather N, Gold R, Hall W (1992). Development of a short 'readiness to change' questionnaire for use in brief, opportunistic interventions among excessive drinkers. Br J Addict.

[36] Witmer Bg, Singer Mj (1998). Measuring presence in virtual environments: a presence questionnaire. Presence.

[37] Kennedy RS, Lane NE, Berbaum KS, Lilienthal MG (1993). Simulator sickness questionnaire: an enhanced method for quantifying simulator sickness. Int J Aviation Psychol.

[38] Dong G, Potenza MN (2014). A cognitive-behavioral model of Internet gaming disorder: theoretical underpinnings and clinical implications. J Psychiatr Res.

[39] Uddin LQ, Iacoboni M, Lange C, Keenan JP (2007). The self and social cognition: the role of cortical midline structures and mirror neurons. Trends Cogn Sci.

[40] Homer BD, Solomon TM, Moeller RW, Mascia A, DeRaleau L, Halkitis PN (2008). Methamphetamine abuse and impairment of social functioning: a review of the underlying neurophysiological causes and behavioral implications. Psychol Bull.

[41] Hui BP, S Wu AM, Pun N (2019). Disentangling the effects of empathy components on Internet gaming disorder: a study of vulnerable youth in China. J Behav Addict.

[42] Windle M, Windle RC (1996). Coping strategies, drinking motives, and stressful life events among middle adolescents: associations with emotional and behavioral problems and with academic functioning. J Abnorm Psychol.

[43] Lidstone JS, Meins E, Fernyhough C (2010). The roles of private speech and inner speech in planning during middle childhood: evidence from a dual task paradigm. J Exp Child Psychol.

