# Supplementary material for: Managing Game-Related Conflict With Parents of Young Adults With Internet Gaming Disorder: Development and Feasibility Study of a Virtual Reality App
Source: JMIR Serious Games. 2021 Jan 18;9(1):e22494. doi: 10.2196/22494 (PMC7850909; doi:10.2196/22494)
Supplement: Multimedia Appendix 1 [file games_v9i1e22494_app1.docx]

Multimedia Appendix 1. Content of participants’ anger expression and virtual parents’ feedback.

| Conflict with the Virtual Mother | |
| --- | --- |
| Suppression | Continues on with the next scene after five seconds of silence |
| Mother’s Feedback | “Why are you not answering me? I know you don’t want to hear me saying this to you, but enough is enough. Why are you always busy playing games instead of studying?” |
| Expression | “Okay, okay. I will turn it off really soon. I haven’t even played for long. Give me a break.” |
| Mother’s Feedback | “Don’t you dare talk back to me. You are always playing that game when you should be focusing on your studies.” |
|  | |
| Conflict with the Virtual Father | |
| Suppression | Continues on with the next scene after five seconds of silence |
| Father’s Feedback | “Why are you not answering me? You have to pull yourself together.” |
| Expression | “Okay, okay. I will turn it off right after another round. You stay out of my business.” |
| Father’s Feedback | “Don’t you think I know you have been playing this game for a really long time? Turn it off right now.” |
